# The effects of different instruments and suture methods of conization for cervical lesions

**DOI:** 10.1038/s41598-019-55786-4

**Published:** 2019-12-13

**Authors:** Xiaoyu Wang, Lei Li, Yalan Bi, Huanwen Wu, Ming Wu, Jinghe Lang

**Affiliations:** 1 Department of Obstetrics and Gynecology, Peking Union Medical College Hospital, Beijing, 100730 China; 2 Department of Pathology, Peking Union Medical College Hospital, Beijing, 100730 China

**Keywords:** Surgical oncology, Urogenital reproductive disorders

## Abstract

This study is to compare the surgical outcomes of patients undergoing cold knife conization (CKC) versus electrosurgical conization (ESC). Among 10,086 patients in a single center admitted between January 2000 and January 2019, CKS or ESC was used for grade 3 cervical intraepithelial neoplasia (CIN3) or more severe lesions. Modified Sturmdorf or Figure-of-eight sutures were applied after conization. A regression model was used to determine the risk factors for margin involvement and short-term post-operative complications. In total, 7275 (72.1%) and 2811 (27.9%) patients underwent CKC and ESC, respectively. Women who underwent ESC were older and had a higher risk of margin involvement and endocervical glandular involvement than those who underwent CKC in univariate analysis. However, in the multivariate analysis, age (odds ratio [OR] 1.032, 95% confidence interval [95% CI] 1.025–1.038) and glandular involvement (OR 2.196, 95% CI 1.915–2.517) were the independent risk factors associated with margin involvement, but the incision methods used caused no significant difference. Modified Sturmdorf sutures and Figure-of-eight sutures were applied in 3520 (34.9%) and 6566 (65.1%) patients, respectively. The modified Sturmdorf sutures was the only risk factor associated with wound hemorrhage (OR 1.852, 95% CI 1.111–3.085) after adjusted with other epidemiological and surgical factors. Various incision or suture methods had similar risk of cervical stenosis. Therefore, ESC is an acceptable alternative to CKC for the diagnosis and treatment of cervical lesions regarding the pathologic accuracy and integrity, and short-term safety. Modified Sturmdorf sutures increased the risk of wound hemorrhage compared with Figure-of-eight sutures.

## Introduction

Cervical cancer is the fourth most frequent cancer in women with an estimated 570,000 new cases in 2018 representing 6.6% of all female cancers^1^. Cervical cancer develops from a multi-stages progression of epithelial cellular changes from persistent oncogenic human papillomavirus (HPV) infection to cervical intraepithelial neoplasia (CIN), a precancerous disease^2^. The prevalence of grade 1 CIN (CIN1), CIN2 and CIN3 in the Chinese population is 3.1%, 1.5% and 1.2%, respectively^3^. Cervical conization is a standard surgical procedure for the diagnosis and treatment of cervical dysplasia and early stage cancer. Currently, in China, cold knife conization (CKC) is used as a primary choice. Alternative methods, such as electrosurgical conization (ESC)^4^, laser conization^5^, harmonic scalpel^6^, and the loop electrosurgical excision procedure (LEEP)^7^, were subsequently developed and attempted. Between different treatment regimens, there was very low quality evidence for the comparison of important parameters, including reproductive outcomes and complications^8^, in particular for screen-and-treat strategies relevant to low- and middle-income countries^9^. Several reports focusing on the comparison between LEEP and CKC even reached controversial conclusions about their effectiveness in the treatment of cervical lesions^10–13^. Specimens obtained by CKC were 50% larger and 100% heavier than the specimens obtained by LEEP^14^. Except for limited specimen volumes for pathological evaluation, high risk of margin involvement, interpretability of the resection margins and tissue fragmentation are other common problems associated with LEEP when utilized in CIN3 or more severe cervical lesions^15^. In such situations, ESC may be an acceptable alternative to CKC. Considering its convenience, few blood loss, wide range of incision sites and low incidence of postoperative hemorrhage^16^, ESC has been widely used in our center. However, doubts about the margin status and other surgical and pathological effects in ESC remained to be clarified.

A paucity of studies concerned CKC versus ESC, and limited information was available regarding the differences in incision margins, pathology and complications. Furthermore, which suture method to choose after conization for cervical shaping and hemostasis was still unknown. The present study aimed to describe the clinicopathological characteristics and postoperative complications between various conization and suture methods in a large cohort of patients from a single study center.

## Materials and Methods

### Ethical approval

This was a cross-sectional study implemented in a tertiary teaching hospital. The Institutional Review Board from the study center approved the study (No. S-K777). All patients provided consents before treatment, and provided informed consent for study participation. The registration number is NCT03961178 (*clinicaltrials.gov*). All procedures performed in the study involving human participants were in accordance with the ethical standards of the institutional and National Research Committee, and with the 1964 *Declaration of Helsinki* and its later amendments or comparable ethical standards. For two patients of 17 years old, informed consents from their parents for study participation were given.

### Study design and patient enrollment

Detailed surgical and pathological data were collected by searching electronic medical records from January 2000 to January 2019. The initial diagnosis was performed based on standard methodology, including cervical cytology followed by colposcopy, with biopsies directed to suspect areas. The inclusion criteria consisted of the following: definite histological results (biopsy and/or conization) CIN3, AIS, stage IA1 squamous cell cervical cancer, or invasive cancer according to FIGO 2009 staging system^17^; and surgeries belonging to CKC or ESC. Patients were excluded if she had insufficient information in their medical records, had no pathological material to review, or if the definite histological diagnosis was CIN1 or CIN2.

### Surgical procedures and follow-up

The excision of a cone-shaped portion of the cervix was performed to remove the cervical lesion and the entire transformation zone by CKC or ESC, which was performed depending on the surgeon’s preference. Iodine was used to smear the cervix before excision to determine the scope of surgery. The extents of the excisions were adjusted according to the nature and extent of the disease, and the purpose of conization (for definite staging or for treatment of CIN). A 3–5 mm ectocervical and endocervical resection margins were typically used. Specifically, the cone height represented the length of the tissue removed from the endocervical canal and was dependent on the deep endocervical extensions of the lesion, the patient’s desires regarding childbearing, and the visibility of the transformed region.

**Figure 1:**
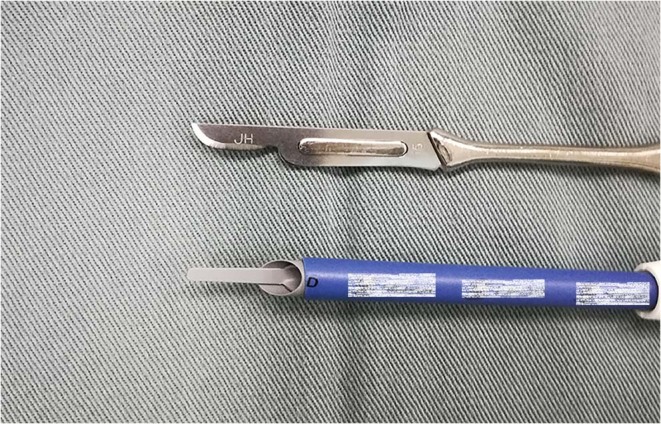
Schemas of the cold knife (up) and electrosurgical instrument (down). The trademarks have been marked.

CKC utilized the 12# or 14# ball cutter, while ESC utilized monopolar electrical cautery commonly used for open surgeries (Fig. 1). These two methods shared similar coning procedures. During the cause of incision, the ESC applied electrocision rather than electrocoagulation to avoid charring the margin of the specimens. Modified Sturmdorf sutures or Figure-of-eight sutures were applied after conization using a #1 absorbable ligature. The former method was operated as described in previous literature^18^. Figure-of-eight sutures were placed on both sides of the conized cervix: a suture was placed in the sequence of 2, 5, 1 and 4 o’clock on the right side, and was placed in the sequence of 10, 7, 11 and 8 o’clock on the left side. Tela iodoform (a prepared thin gauze dipped in iodine tincture and glycerinum) was placed into the cervical canal to avoid hemorrhage and infection, and was removed 7 days after conization in both methods. In the study center, all the surgeons had their own discretion of conization and suture methods. Many surgeons, such as the authors (LL and MW), insisted on a certain surgical and/or suture method all the time; other surgeons in our center would attempt or even change their practice after a certain period.

The first follow-up occurred with 6 weeks after the surgery, which provided an opportunity of discussing pathology and examining the cervical sutures. Later, in the first year after surgery, a follow-up was provided at an interval 3 to 6 months; in the second year and later, a follow-up was provided at an interval 6 to 12 months and at each year, respectively. The follow-up consisted of testing of high risk HPV and cytology. The management of any abnormal findings followed the current guideline^19^.

Short-term postoperative complications were collected by reviewing patient inpatient and emergency room records. Wound hemorrhage and cervical stenosis were the most commonly observed complications and received attention. In this report, wound hemorrhage was defined as situations with at least one of the following conditions documented in case reports: estimated blood loss greater than 200 ml during the conization surgery; transfusion during or within 7 days after the surgery; and vaginal or retroperitoneal hemorrhage needing surgical or interventional treatment. Cervical stenosis was defined as situations with at least one of the following conditions requiring interventions: amenorrhea with hematometra after the surgery and no passing of the uterine sound through the cervix; longer menstruation periods than preoperative conditions with lower abdominal pain, fever or endometrial cavity fluid. Other less common short-term complications during and/or after the conization included injuries to the bladder, ureter or bowel, retroperitoneal hematoma, and severe infections.

### Pathological assessment

The cone specimens were cut apart from the anterior lips at the 12 o’clock position, fixed in 10% formalin, and embedded in paraffin. Twelve 4-μm-thick serial sections were stained with hematoxylin-eosin (HE) and examined. Pathological information was collected from the pathology reports. Trichotomy nomenclature was used for the precursors of cervical tumors (CIN1–3) in the study center. Attention was paid to the cone height and diameter, margin status, endocervical glandular involvement, disease nature (cancer or CIN) and lymph-vascular space invasion (LVSI). Any patient with missing or ambiguous information was reviewed by two pathologists (HW and YB). The diagnosis of carcinoma *in situ* and invasive cancer before 2010 were also reviewed and modified according to FIGO 2009 system. Cone height and diameter were measured by pathologists rather than by surgeons, as most specimens had not been accurately described in the surgical reports. Margin status (endocervical, ectocervical) were defined as positive based on the presence of AIS, invasive carcinoma or less than 1 mm at the edge of the specimens, or on the presence of CIN or less than 0.1 mm. Endocervical glandular involvement was defined as dysplastic squamous epithelium occupying well-circumscribed, rounded spaces in the depth of the cervical stroma^20^.

### Statistical analysis

Comparisons of continuous variables were conducted using parametric methods if assumptions of normal distribution were confirmed. Nonnormally distributed variables and categorical data were compared among the various groups using nonparametric tests. The logistic regression method was used to analyze the risk factors associated with margin involvement and postoperative complications (cervical stenosis and wound hemorrhage). An odds ratio (OR) and the 95% confidence interval (95% CI) were obtained in the multivariate model using significant clinicopathological factors. Unless otherwise stated, all analyses were performed with a two-sided significance level of 0.05 using SPSS 22.0 (SPSS, Inc., Chicago, IL, USA).

### Ethics approval and registration

The Institutional Review Board of Peking Union Medical College Hospital has approved this study (No. S-K777). The registration number is NCT03961178 (*clinicaltrials.gov*).

### Condensation

Electrosurgical conization is an acceptable method for CIN3 or more severe cervical lesions, and Figure-of-eight suture provides secure wound closing.

## Results

### Demographic data of the study population

**Figure 2:**
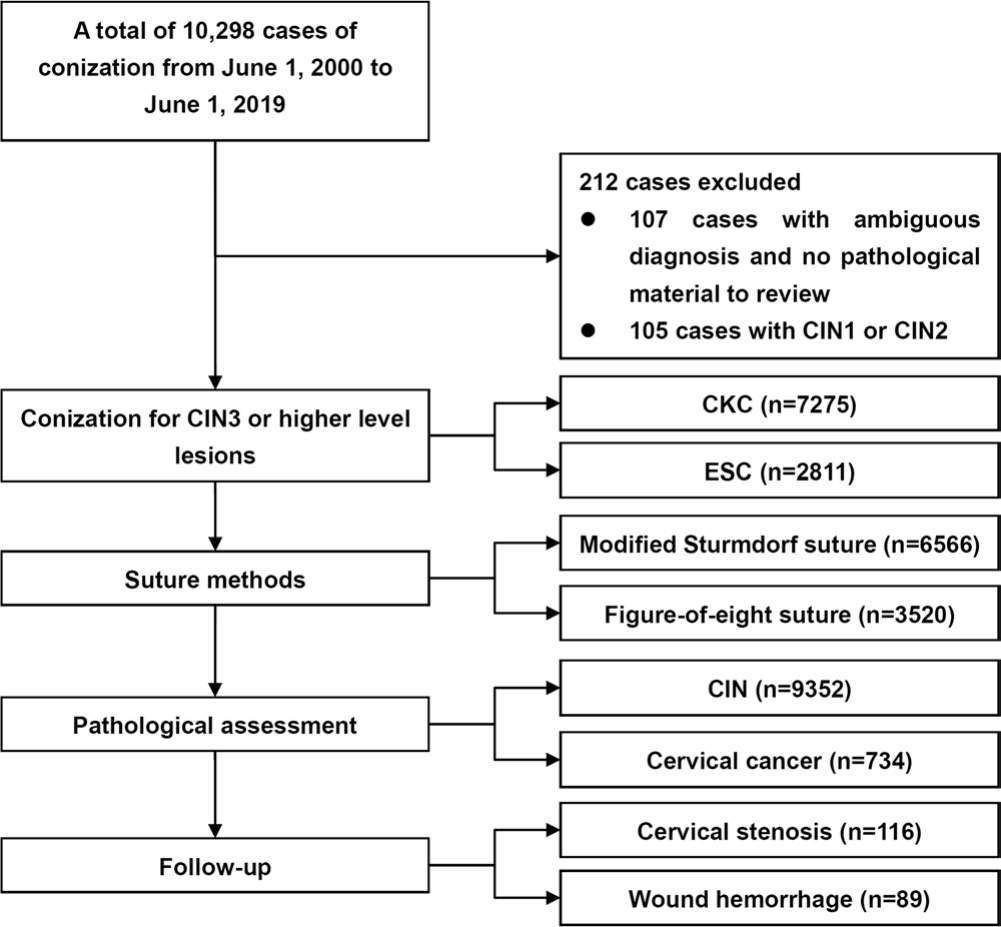
The flow diagram of the study. CKC, cold knife conization. ESC, electrosurgical conization. CIN, cervical intraepithelial neoplasia.

**Figure 3:**
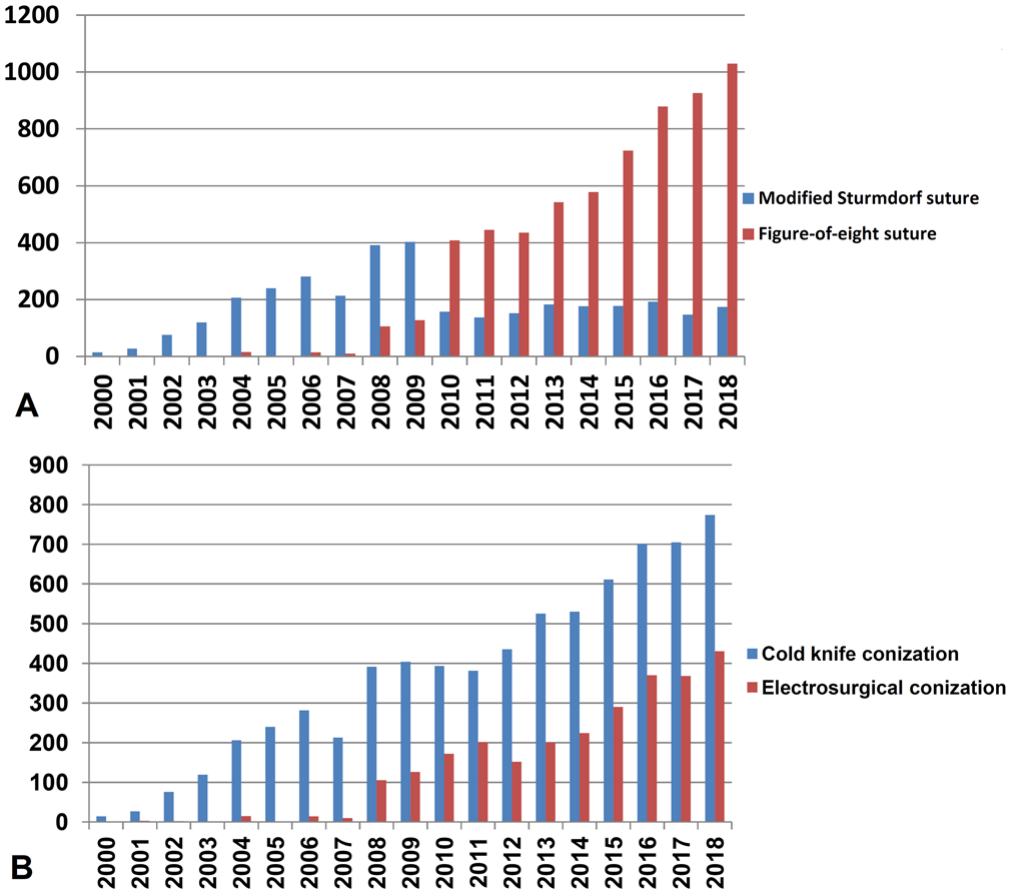
The trend of cases of various conization and suture methods in each year. As this study only included cases in January of 2019, the data of 2019 was not displayed. (**A**) Trend of various suture methods in each year. (**B**) Trend of various conization methods in each year.

A total of 10,086 patients were included in the retrospective study (Fig. 2, Table S1), and the trends in various conization and suture methods are displayed in Fig. 3. The median age in the series was 39 years (range, 17–85). In total 1391 patients (13.8%) were in menopausal status. CKC and ESC were performed in 7275 (72.1%) and 2811 (27.9%) patients, respectively (Table 1). The median height and diameter of the cone were 16 mm (range, 10–35) and 27.95 mm (range, 14–42), respectively. Figure-of-eight sutures and modified Sturmdorf sutures were applied in 6566 (65.1%) and 3520 (34.9%) patients, respectively.

**Table 1:** Comparison for clinical characteristics of patients in CKC and ESC groups by univariate analysis. CKC, cold knife conization.

	CKC (n = 7275)	ESC (n = 2811)	*p*
Age (year), range (medium)	39 (17–85)	40 (17–73)	< 0.001
Cone height (mm), range (medium)	16 (10–35)	16 (10–35)	< 0.001
Cone diameter (mm), range (medium)	27.95 (14–42)	28.22 (14–42)	0.011
Margin involvement, n (%)	1045 (14.4%)	474 (16.9%)	0.002
Endocervical glandular involvement, n (%)	4637 (63.7%)	1898 (67.5%)	< 0.001
**Disease nature, n (%)**			< 0.001
Cancer	472 (6.5%)	262 (9.3%)	
CIN	6803 (93.5%)	2549 (90.7%)	
**Stages of squamous carcinoma, n (%)**			0.423
IA1	92 (29.1%) (n = 316)	41 (25.6%) (n = 160)	
>IA1	224 (70.9%) (n = 316)	119 (74.4%) (n = 160)	
**Stages of adenocarcinoma, n (%)**			0.162
*In situ*	98/148 (65.8%)	57/100 (57.0%)	
Invasive	51/149 (34.2%)	43/100 (43.0%)	
Invasion depth of cancer (mm), range (medium)	4 (1–21) (n = 346)	4 (1–20) (n = 192)	0.440
Invasion width of cancer (mm), range (medium)	7 (1–40) (n = 260)	7 (1–23) (n = 140)	0.332
LVSI, n (%)	101 (27.0%) (n = 374)	49 (23.9%) (n = 205)	0.415

ESC, electrosurgical conization. CIN, cervical intraepithelial neoplasia. LVSI, lymph-vascular space invasion.

Values are given as median (range) or number (percentage).

Pathological evaluation revealed CIN3 in 9352 patients (92.7%) and cervical cancer in 734 patients (7.3%), which included 155 AISs, 93 adenocarcinomas, 476 squamous cell carcinomas (SCCs), 5 adenosquamous carcinomas, 4 clear cell carcinomas and 1 small cell carcinoma. In the patients with SCCs, 133 of 476 patients (27.9%) belonged to stage IA1. For invasive cervical cancer, the median depth and width of the cancer lesion were 4 mm (range, 0.5–11) and 7 mm (range, 0.5–40), respectively. Positive LVSI appeared in 150 of 579 cancer patients (25.9%). Thirty-two patients (0.3%) had concurrent vaginal lesions, including 27 with vaginal squamous intraepithelial lesions (VaINs) and 5 with vaginal cancer. Eight patients (0.08%) also had concurrent vulvar lesions, including 5 with vulvar squamous intraepithelial lesions (VINs) and 3 with vulvar cancer. The endocervical endocervical glandular involvement was found in 6536 (64.8%) patients.

As shown in Fig. 3, CKC and ESC increased with years, but more surgeries have applied Figure-of-eight sutures recently. Among 7275 patients who received CKC, 3520 underwent modified Sturmdorf sutures, and none of 2811 patients who received ESC underwent modified Sturmdorf sutures (*p* < 0.001). Regarding the different suture methods, patients with modified Sturmdorf sutures and Figure-of-eight sutures had similar epidemiological characteristics, except the patients in former group were significantly younger than the patients in the latter group (median 39 [range 19–85] versus 40 [17–83] years, *p* < 0.001).

The clinical characteristics of the CKC and ESC groups are displayed in Table 1. In univariate analysis, age, cone height, cone diameter, margin involvement, endocervical glandular involvement and disease nature (CIN or cancer) were significantly different between the two groups. Women with CKC were significantly younger than women with ESC (*p* < 0.001). Cone specimens after ESC had a higher height (*p* < 0.001) and larger diameter (*p* = 0.011) but more chances of margin involvement (*p* = 0.002) and glandular involvement (*p* < 0.001). Pathological results showed that cervical cancer represented a high proportion in the ESC group (*p* < 0.001).

### Margin involvement and its risk factors

The status of margin involvement is shown in Table 2. A positive surgical margin was found in 1519 (15.1%) patients. Margin involvement presented in 1122 of 9352 CIN patients (12.0%) and 397 of 734 cervical cancer patients (54.1%), respectively. In all 1519 patients, endocervical, ectocervical and both endo- and ectocervical involvement occurred in 701 (46.1%), 601 (39.6%) and 217 cases (14.3%), respectively.

**Table 2:** Comparison of margin involvement between CKC and ESC groups.

	All patients			Cervical Cancer			CIN		
	CKC	ESC	*p*	CKC	ESC	*p*	CKC	ESC	*p*
Margin involvement			0.002			0.413			0.133
Positive	1045 (14.4%)	474 (16.9%)		250 (53%)	147 (56.1%)		795 (11.7%)	327 (12.8%)	
Negative	6230 (85.6%)	2337 (83.1%)		222(47%)	115 (43.9%)		6008 (88.3%)	2222 (87.2%)	
Positive margin status			0.001			0.161			0.008
Endocervical	453 (43.3%)	248 (52.3%)		96 (38.4%)	69 (46.9%)		357 (44.9%)	179 (54.7%)	
Ectocervical	445 (42.6%)	156 (32.9%)		93 (37.2%)	42 (28.6%)		352 (44.3%)	114 (34.9%)	
Endo + ectocervical	147 (14.1%)	70 (14.8%)		61 (24.4%)	36 (24.5%)		86 (10.8%)	34 (10.4%)	

CKC, cold knife conization. ESC, electrosurgical conization. CIN, cervical intraepithelial neoplasia.

Among all the patients in the series, regardless of whether they were analyzed at a whole (*p* = 0.001) or separately according to different margins (*p* = 0.002), ESC was likely to induce positive margins than CKC in univariate analysis (Table 2). However, for patients with cervical cancer, the differences were not statistically significant. For patients with CIN, ESC was more likely to induce inner margin involvement (*p* = 0.008).

Clinicopathological factors associated with margin involvement in multivariate model were listed in Table 3. In the total series, the multivariate analysis by logistic regression showed that age, endocervical glandular involvement and disease nature were associated with the involvement of positive surgical margins. For patients with cervical cancer, age was the only risk factor associated with margin involvement. For patients with CIN, the risk factors were age and endocervical glandular involvement. In the whole cohort, the conization method, cone height and cone diameter were not risk factors associated with margin involvement (Table 3).

**Table 3:** Clinicopathological factors associated with margin involvement in multivariate model.

	All patients		Cervical cancer		CIN	
	*p*	OR (95% CI)	*p*	OR (95% CI)	*p*	OR (95% CI)
Age	<0.001	1.035 (1.025–1.044)	<0.001	1.078 (1.052–1.105)	<0.001	1.027 (1.018–1.037)
Menopausal	0.398	1.098 (0.884–1.365)	0.145	1.528 (0.864–2.700)	0.705	1.047 (0.825–1.329)
Conization method	0.207	1.086 (0.956–1.233)	0.155	1.261 (0.916–1.736)	0.400	1.062 (0.923–1.222)
Cone height	0.080	0.991 (0.980–1.001)	0.903	1.002 (0.974–1.030)	0.059	0.989 (0.978–1.000)
Cone diameter	0.517	1.002 (0.995–1.009)	0.351	1.009 (0.990–1.027)	0.841	1.001 (0.993–1.008)
Endocervical glandular involvement	<0.001	2.192 (1.912–2.513)	0.416	1.134 (0.837–1.538)	<0.001	2.635 (2.244–3.095)
Disease nature	<0.001	9.760 (8.251–11.545)	N/A	N/A	N/A	N/A

CIN, cervical intraepithelial neoplasia. OR, odds ratio. 95% CI, 95% confidence interval. N/A, not available.

### Postoperative complications and their risk factors

A total of 207 (2.0%) patients suffered from surgical complications, including 89 (0.9%) patients with wound hemorrhage,116 (1.2%) with cervical stenosis, and 6 (0.06%) patients admitted for severe adverse events associated: two patients with bladder injuries (one each in the CKC and ESC groups), one patient with a unilateral ureter injury (in the CKC group), one patient with a retroperitoneal hematoma (in the CKC group), and two patients with septic shock (both in the CKC group). No bowel injuries were documented.

In univariate analysis, between the CKC and ESC groups, there were no statistically significant differences in cervical stenosis (87 and 29 patients, 1.2% vs 1.0%, *p* = 0.488) or wound hemorrhage (64 and 25 patients, 0.9% versus 0.9%, *p* = 0.963). However, patients with modified Sturmdorf sutures had a higher risk of wound hemorrhage than patients with Figure-of-eight sutures (40 and 49 patients, respectively, 1.1% vs 0.9%, *p* = 0.046) but not cervical stenosis (47 and 69 patients, respectively, 1.3% versus 1.1%, *p* = 0.202).

The multivariate analysis using parameters of age, cone height, cone diameter, and conization and suture method revealed that, modified Sturmdorf suture was the only independent risk factor associated with wound hemorrhage (OR 1.852, 95% CI 1.111–3.085, *p* = 0.018) but not cervical stenosis (OR 1.286, 95% CI 0.840–1.969, *p* = 0.248). No independent factor was found relevant to cervical stenosis in this model.

## Discussion

### Principal Findings

Our study provides evidence of the effects of CKC and ESC on conization. Although ESC was associated with more margin involvement, the difference was mainly caused by a larger number of patients with cancer and older patients in this group compared with CKC group, and the difference lost significance in the multivariate model. In addition, ESC and CKC resulted in the same incidence of short-term complications. These findings support ESC as an acceptable alternative to CKC in women for the diagnosis and treatment of CIN3 or more severe cervical lesions. The electrosurgical unit produces a continuous wave for the cutting current and a discontinuous wave for the coagulation current, which could shorten the operation time and overcome the drawbacks of CKC in terms of hemostasis^21^. Monopolar electrosurgical equipment exists widely, even in low-resource clinical settings, and the manipulations are easily performed without special instruments or trainings. Such convenience may shorten the learning curve of conization.

In the present study, positive surgical margins were found in 15.1%, 12% and 54.1% of the total, CIN and cancer patients, respectively. The lower prevalence in our study might be explained by the wide surgical scope. The high incidence of involved margin in cancer patients was mainly attributing to the diagnostic nature of conization in such situations. A meta-analysis of 35,109 patients with any grade CIN or invasive cancer found that 23% had positive surgical margins^22^. The rate of positive surgical margin involvement in the LEEP varied from 22% to 33.8%, in CKC from 13% to 22.3%, and in ESC from 4.7% to 25.7%^10,21,23–25^. It has been confirmed that a conization depth less than 10 mm might be a risk factor that predicts endocervical resection margins and had the highest sensitivity (100%) among the other factors including cone volume and cone base surface^26,27^. In our study, although the height and diameter of the cone between the CKC and ESC procedures were significantly different, a cone height of at least 10 mm was reached for each patient. Therefore, neither cone height nor cone diameter was a risk factor associated with margin involvement.

There were other important issues to be considered and balanced before wide utilization of ESC. It has been proposed that the monopolar system of ESC could lead to more tissue fragments, similar to LEEP^23^. If it was true, the pathological evaluation of ESC specimens should have lost many important features, such as margin status, LVSI, invasion depth and width. However, in our study, most of these pathological parameters were similar between CKC and ESC groups. ESC even revealed more patients with margin and endocervical glandular involvement, and more patients with cancer or invasive cancer (Table 1). Besides, in a randomized controlled study, Camargo *et al*.^28^ found that straight-wire excision of the transformation zone and large loop excision of the transformation zone were equally effective in treating endocervical disease, with no difference in protecting against margin involvement. Higher, but not severe, blood loss and longer surgical time were observed in the former group^28^. These findings support the direct utilization of electrosurgical procedures in conization.

In our study, only age and endocervical glandular involvement were independent risk factors for margin involvement in the multivariate model, especially in patients with CIN. The relationship between age and residual disease had been validated by published studies^29^. These finding might be due to the migration of the transformation zone to the cervical canal with increased age and following menopause, which leads to difficulties in removing the whole transformation zone in elderly and postmenopausal women^30,31^. The significance of endocervical glandular involvement in the pathological evaluation of cervical lesions is controversial. Endocervical glandular involvement was reported to be four times more common in a high-grade squamous intraepithelial lesion (HSIL) compared with an LSIL^32^; there is approximately 17–25% endocervical glandular involvement in CIN1 and 81–91% endocervical glandular involvement in CIN3^20,33^. However, it is still controversial whether or not endocervical glandular involvement was associated with HSIL recurrence^29,34,35^. These findings support long-term follow-up in the treatment and prognosis of CIN to reveal the role of endocervical glandular involvement in the pathological evaluation of conization specimens.

Wound hemorrhage occurred in 0.9% of patients during and/or short-term after conization in our series, and the modified Sturmdorf suture increased the hemorrhage risk. Several studies have suggested that suturing after conization could prevent early and late hemorrhage compared with electrosurgical procedures^36,37^. The hemorrhage issue of CKC with Sturmdorf sutures had been questioned before^38^. However, limited studies have compared the relationship between suture methods and postoperative hemorrhage, and no study has reported the utilization of Figure-of-eight sutures in conization until now. In other reports, the Figure-eight technique was employed for muscle laceration repairs^39^, venous hemostasis^40^, and vaginal cuff closure^41^ due to its simplicity and efficiency. In our study, Figure-of-eight sutures resulted in few incidences of hemorrhage according to our definition than modified Sturmdorf sutures, most likely because the former provided more secure closing of residual cervical tissues. The genuine effects of various suture methods on hemorrhage during and after conization should be tested in a well-designed randomized controlled study.

Cervical stenosis occurred in 1.2% of patients after conization in our series. Due to the retrospective design and the definition of cervical stenosis in our study, the actual incidence was probably underestimated, as most menopausal patients had no or less complaint of cervical stenosis. In the published literature, the incidence of cervical stenosis varies across studies from 1% to 15% depending on the definition employed^42,43^. Reported risk factors for cervical stenosis include elderly patients (aged ≥ 46 years)^44^ and deep incisions^45^. However, in our study, age, cone height and width, conization and suture methods had no significant impact on the incidence of cervical stenosis. The placement of tela iodoform, the underestimated incidences, and relative uniform incision depth were probably reasons that could explain the difference between our conclusion and previous reports.

In our practice reported in this study, large incision width and height were typically used, which would be wide enough even for adenocarcinoma *in situ* or stage IA1 cancer. Whether a reduced incision width for CIN3 was appropriate or not deserves further exploration, as such caution was probably associated with decreased risk of preterm^46^. Although specimens obtained by CKC were larger and heavier than the specimens obtained by LEEP^14^, the potential productive risks deserve cautious practice and individual management.

The large sample size and comprehensive pathological description were the main strengths of our study. There were several limitations to our work. The most important one is the observational nature of the data. In particular, two conization devices were used over a span of 19 years, leading to a selection bias. In addition, as involved surgeons had different references, the surgical technique was not uniform. It was reported the expertise of the gynecologic surgeon who performed the conization procedures appeared to influence the rate of involved margins after conization^47^. Another limitation of this study is the lack of prognostic and fertility outcome analyses, since incomplete excision of cervical precancer as a predictor of treatment failure^48^, and adverse obstetric outcomes after local treatment for cervical lesions increased according to cone depth^49^. A further extensive follow-up of this study and a well-designed randomized clinical trial is required to compare the recurrence and obstetric outcome of CKC and ESC in the management of cervical lesions. A trichotomy rather than a dichotomy nomenclature (LSIL and HSIL) utilized in our center was another methodological limitation, which could probably prevent the generalization of our findings. Last, several studies had revealed the relationship between margin status, recurrence and smoking status or numbers (sweeps) of specimen in the treatment of CIN by conization or by LEEP^22,50–52^. However, due to the retrospective nature of our study, we couldn’t clearly state these potential risk factors, which would possibly limit our findings.

## Conclusions

ESC is an alternative technique of CKC for the diagnosis and treatment of CIN3 or cervical cancer, with identical pathological description and surgical complications. Old patients and those with endocervical glandular involvement should be concerned with margin involvement and need close follow-up. Modified Sturmdorf sutures have more chances to induce wound hemorrhage compared with Figure-of-eight methods.

## Supplementary information

STROBE checklist

Legend of Supplementary Table

Table S1

## References

[1] World Health Organization, Cervical cancer. https://www.who.int/cancer/prevention/diagnosis-screening/cervical-cancer/en/. Accessed on November 24, 2019.

[2] Cohen PA (2019). Cervical cancer. Lancet..

[3] Shi JF (2012). The burden of cervical cancer in China: synthesis of the evidence. Int J Cancer..

[4] Lee SJ (2009). Conization using electrosurgical conization and cold coagulation for international federation of gynecology and obstetrics stage IA1 squamous cell carcinomas of the uterine cervix. Int J Gynecol Cancer..

[5] Akiba Y (2005). Is laser conization adequate for therapeutic excision of adenocarcinoma *in situ* of the uterine cervix?. J Obstet Gynaecol Res..

[6] Kartsiounis C (2011). Comparison of the ultrasonic scalpel to CO(2) laser in cervical conization. Minim Invasive Ther Allied Technol..

[7] Chen L (2019). Risk Factor Analysis of Persistent High-Grade Squamous Intraepithelial Lesion After Loop Electrosurgical Excision Procedure Conization. J Low Genit Tract Dis..

[8] Santesso N (2016). Systematic reviews and meta-analyses of benefits and harms of cryotherapy, LEEP, and cold knife conization to treat cervical intraepithelial neoplasia. Int J Gynaecol Obstet..

[9] Santesso N (2016). World Health Organization Guidelines for treatment of cervical intraepithelial neoplasia 2-3 and screen-and-treat strategies to prevent cervical cancer. Int J Gynaecol Obstet..

[10] Panna S (2009). Positive margin prevalence and risk factors with cervical specimens obtained from loop electrosurgical excision procedures and cold knife conization. Asian Pac J Cancer Prev..

[11] El-Nashar SA (2017). Loop Electrosurgical Excision Procedure Instead of Cold-Knife Conization for Cervical Intraepithelial Neoplasia in Women With Unsatisfactory Colposcopic Examinations: A Systematic Review and Meta-Analysis. J Low Genit Tract Dis..

[12] Zeng SY (2012). Comparison of the efficacy and complications of different surgical methods for cervical intraepithelial neoplasia. Eur J Gynaecol Oncol..

[13] Munro A (2015). Comparison of cold knife cone biopsy and loop electrosurgical excision procedure in the management of cervical adenocarcinoma *in situ*: What is the gold standard?. Gynecol Oncol..

[14] Fanning J (2002). Cold knife conization vs. LEEP. Are they the same procedure?. J Reprod Med..

[15] Malapati R (2011). Factors influencing persistence or recurrence of cervical intraepithelial neoplasia after loop electrosurgical excision procedure. J Low Genit Tract Dis..

[16] Akazawa M (2019). Comparison of Electrosurgical Devices for Cervical Conization: Novel Monopolar Scalpel (VIO) Versus Ultrasonic Scalpel. J Low Genit Tract Dis..

[17] Pecorelli S (2009). Revised FIGO staging for carcinoma of the vulva, cervix, and endometrium. Int J Gynaecol Obstet..

[18] Bielecki A (1964). Modification of the Suture in the Sturmdorf Operation. Am J Obstet Gynecol..

[19] Saslow D (2012). American Cancer Society, American Society for Colposcopy and Cervical Pathology, and American Society for Clinical Pathology screening guidelines for the prevention and early detection of cervical cancer. J Low Genit Tract Dis..

[20] Kir G (2012). Endocervical glandular involvement, positive endocervical surgical margin and multicentricity are more often associated with high-grade than low-grade squamous intraepithelial lesion. J Obstet Gynaecol Res..

[21] Xiang L (2015). Conization Using an Electrosurgical Knife for Cervical Intraepithelial Neoplasia and Microinvasive Carcinoma. PLoS One..

[22] Ayhan A (2016). Risk factors for residual disease after cervical conization in patients with cervical intraepithelial neoplasia grades 2 and 3 and positive surgical margins. Eur J Obstet Gynecol Reprod Biol..

[23] Wang XI (2017). Loop Electrosurgical Excision Procedure vs. Cold Knife Cone in Treatment of Cervical Intraepithelial Neoplasia: Review of 447 Cases. Ann Clin Lab Sci..

[24] Jiang YM (2016). Meta-analysis of cold-knife conization versus loop electrosurgical excision procedure for cervical intraepithelial neoplasia. Onco Targets Ther..

[25] Kigure K (2018). An electrical scalpel conization versus Shimodaira-Taniguchi conization procedure for cervical intraepithelial neoplasia. Medicine (Baltimore)..

[26] Papoutsis D (2011). Cervical cone measurements and residual disease in LLETZ conisation for cervical intraepithelial neoplasia. In Vivo..

[27] Papoutsis D (2013). Appropriate cone dimensions to achieve negative excision margins after large loop excision of transformation zone in the uterine cervix for cervical intraepithelial neoplasia. Gynecol Obstet Invest..

[28] Camargo MJ (2015). Large loop versus straight-wire excision of the transformation zone for treatment of cervical intraepithelial neoplasia: a randomised controlled trial of electrosurgical techniques. BJOG..

[29] Park JY (2007). Risk factors predicting residual disease in subsequent hysterectomy following conization for cervical intraepithelial neoplasia (CIN) III and microinvasive cervical cancer. Gynecol Oncol..

[30] Bae HS (2013). The appropriate cone depth to avoid endocervical margin involvement is dependent on age and disease severity. Acta Obstet Gynecol Scand..

[31] Papakonstantinou K (2014). Management of stage Ia1 squamous cervical cancer and the importance of excision margins: a retrospective study of long-term outcome after 25 years of follow-up. Am J Obstet Gynecol..

[32] Nagi CS (2006). Endocervical glandular involvement is associated with high-grade SIL. Gynecol Oncol..

[33] Guducu N (2013). Endocervical glandular involvement, multicentricity, and extent of the disease are features of high-grade cervical intraepithelial neoplasia. Ann Diagn Pathol..

[34] Paraskevaidis E (2000). Cervical intraepithelial neoplasia outcomes after large loop excision with clear margins. Obstet Gynecol..

[35] Kodampur M (2013). Endocervical crypt involvement by high-grade cervical intraepithelial neoplasia after large loop excision of transformation zone: do we need a different follow-up strategy?. J Obstet Gynaecol Res..

[36] Dane C (2008). Haemostasis after cold-knife conisation: a randomised prospective trial comparing cerclage suture versus electro-cauterization. Aust N Z J Obstet Gynaecol..

[37] Furugori M (2017). Short- and long-term complications and the impact on quality of life after cervical conization by harmonic scalpel. J Obstet Gynaecol Res..

[38] Kristensen GB (1990). A randomized trial comparing two methods of cold knife conization with laser conization. Obstet Gynecol..

[39] Castillo A. C., *et al*. Comparing 3 Suture Techniques After Muscle Laceration Repair. *Hand (N Y)*. 1558944719837021 (2019). 10.1177/1558944719837021 PMC7818024 30924359

[40] Okada M (2018). Efficacy and Safety of Figure-of-Eight Suture for Hemostasis After Radiofrequency Catheter Ablation for Atrial Fibrillation. Circ J..

[41] Tsafrir Z (2017). Long-term outcomes for different vaginal cuff closure techniques in robotic-assisted laparoscopic hysterectomy: A randomized controlled trial. Eur J Obstet Gynecol Reprod Biol..

[42] Monteiro AC (2008). Cervical stenosis following electrosurgical conization. Sao Paulo Med J..

[43] Nasu K (2010). Management of severe cervical stenosis after conization by detention of nylon threads tied up to intrauterine contraceptive device. Arch Gynecol Obstet..

[44] Tanaka Y (2017). Predictors for recurrent/persistent high-grade intraepithelial lesions and cervical stenosis after therapeutic conization: a retrospective analysis of 522 cases. Int J Clin Oncol..

[45] Hasegawa K (2016). The problems of cervical conization for postmenopausal patients. Eur J Gynaecol Oncol..

[46] Castanon A (2014). Risk of preterm delivery with increasing depth of excision for cervical intraepithelial neoplasia in England: nested case-control study. BMJ..

[47] Ulrich D (2012). Conization of the uterine cervix: does the level of gynecologist’s training predict margin status?. Int J Gynecol Pathol..

[48] Arbyn M (2017). Incomplete excision of cervical precancer as a predictor of treatment failure: a systematic review and meta-analysis. Lancet Oncol..

[49] Kyrgiou M, *et al*. Adverse obstetric outcomes after local treatment for cervical preinvasive and early invasive disease according to cone depth: systematic review and meta-analysis. *BMJ*. i3633 (2016). 10.1136/bmj.i3633 PMC4964801 27469988

[50] de Mello Silva MV (2014). Factors associated with the persistence/recurrence of CIN2/3 in women submitted to loop electrosurgical excision procedure in a teaching hospital in northeastern Brazil: a case-control study. J Low Genit Tract Dis..

[51] Simoes RB (2013). Post-cervical conization outcomes in patients with high-grade intraepithelial lesions. APMIS..

[52] Satmary W (2018). Vulvar intraepithelial neoplasia: Risk factors for recurrence. Gynecol Oncol..

