# Supplementary material for: The effects of different instruments and suture methods of conization for cervical lesions
Source: Sci Rep. 2019 Dec 13;9:19114. doi: 10.1038/s41598-019-55786-4 (PMC6910914; doi:10.1038/s41598-019-55786-4)
Supplement: Supplementary file 2 — Legend of Supplementary Table [file 41598_2019_55786_MOESM2_ESM.docx]

**Title**

The effects of different instruments and suture methods of conization for cervical lesions

**Short title**

Different instruments and sutures for conization

**Authors**

Xiaoyu Wang, M.D.,^1^ yuluosiji@126.com

Lei Li, M.D.,^1^ lileigh@163.com

Yalan Bi, M.D.,^2^ biyeye81@126.com

Huanwen Wu, M.D.,^2^ pumch_gynecology@163.com

Ming Wu, M.D.,^1^ wuming@pumch.cn

Jinghe Lang, M.D., ^1^ langjh@vip.163.com

**Affiliations**

^1^ Department of Obstetrics and Gynecology, Peking Union Medical College Hospital, Beijing 100730, China

^2^ Department of Pathology, Peking Union Medical College Hospital, Beijing 100730, China

**Disclosure**

All authors declare that they have no conflicts of interest to disclose.

**Funding**

This study was supported by the Chinese Academy of Medical Sciences Initiative for Innovative Medicine (CAMS-2017-I2M-1-002). The funders had no role in study design, data collection and analysis, decision to publish, or preparation of the manuscript.

**Ethics approval and registration**

The Institutional Review Board of Peking Union Medical College Hospital has approved this study (No. S-K777). The registration number is NCT03961178 (*clinicaltrials.gov*).

**Corresponding authors**

Lei Li, M.D.

Department of Obstetrics and Gynecology, Peking Union Medical College Hospital

Address: Shuaifuyuan No. 1, Dongcheng District, Beijing 100730, China

Email: lileigh@163.com (LL)

Phone: 86-139-1198-8831

**Supplement legend**

Table S1

Raw data of all patients
